# Functional self-assembling peptide nanofiber hydrogel for peripheral nerve regeneration

**DOI:** 10.1093/rb/rbw034

**Published:** 2016-12-19

**Authors:** Xiaoli Wu, Liumin He, Wen Li, Heng Li, Wai-Man Wong, Seeram Ramakrishna, Wutian Wu

**Affiliations:** 1 Department of Anatomy, School of Biomedical Science, LKS Faculty of Medicine, The University of Hong Kong, 21 Sassoon Road, Hong Kong SAR, China; 2 Department of Anatomy, School of Biomedical Science, LKS Faculty of Medicine, The University of Hong Kong, 21 Sassoon Road, Hong Kong SAR, China; 3 State Key Laboratory of Brain and Cognitive Sciences, Li Ka Shing Faculty of Medicine, The University of Hong Kong, Pokfulam, 21 Sassoon Road, Hong Kong SAR, China; 4 Joint Laboratory of Jinan University and the University of Hong Kong, GHM Institute of CNS Regeneration, Jinan University, 601 Huangpu Avenue West, Guangzhou, 510632, China; 5 Department of Mechanical Engineering, Faculty of Engineering, Center for Nanofibers and Nanotechnology, National University of Singapore, 117576, Singapore, Singapore.

**Keywords:** Peripheral nerve regeneration, self-assembling peptide, IKVAV, RGD, nanofibrous hydrogel

## Abstract

Peripheral nerves are fragile and easily damaged, usually resulting in nervous tissue loss, motor and sensory function loss. Advances in neuroscience and engineering have been significantly contributing to bridge the damage nerve and create permissive environment for axonal regrowth across lesions. We have successfully designed two self-assembling peptides by modifying RADA 16-I with two functional motifs IKVAV and RGD. Nanofiber hydrogel formed when combing the two neutral solutions together, defined as RADA 16-Mix that overcomes the main drawback of RADA16-I associated with low pH. In the present study, we transplanted the RADA 16-Mix hydrogel into the transected rat sciatic nerve gap and effect on axonal regeneration was examined and compared with the traditional RADA16-I hydrogel. The regenerated nerves were found to grow along the walls of the large cavities formed in the graft of RADA16-I hydrogel, while the nerves grew into the RADA 16-Mix hydrogel toward distal position. RADA 16-Mix hydrogel induced more axons regeneration and Schwann cells immigration than RADA16-I hydrogel, resulting in better functional recovery as determined by the gait-stance duration percentage and the formation of new neuromuscular junction structures. Therefore, our results indicated that the functional SAP RADA16-Mix nanofibrous hydrogel provided a better environment for peripheral nerve regeneration than RADA16-I hydrogel and could be potentially used in peripheral nerve injury repair.

## Introduction

Peripheral nerve injuries, which may result in loss of motor function, sensory function, or both, affect 130–230 per 1 million persons each year [1]. Because patients with peripheral nerve injuries are usually at their peak of employment, the loss or decrease in function can be particularly devastating. Despite advancements in microsurgical techniques in the past decades, functional recovery is still unsatisfactory with no complete recovery achieved [2]. Therefore, treatment of peripheral nerve injuries is one of the most challenging modern medical problems. After peripheral nerve injury, Wallerian degeneration acts to clear and provide a regenerative environment [3, 4]. Nervous tissue loss in the injury site makes it difficult for the regenerating axons to re-enter the distal nerve stumps and re-innervate the muscular targets, leading to functional impairment and frequently to neuroma. Furthermore, the slow regeneration rate is detrimental to the functional outcome as well [5]. Hence, strategies to create a supportive and permissive environment are critical for nerve regeneration, including axonal elongation and circuit reestablishment [6, 7]

In most clinical cases, autologous nerve graft is served as the gold standard for the peripheral nerve repair. But autologous nerve graft has been limited to the mismatch size between the donor and host nerve, as well as additional surgery and trauma to the donor nerve [8]. Besides, neuroma can formed within the reconnection site after transplantation [9]. In recent years, synthetic biomaterial scaffolds have been developed as an alternative to nerve autograft with varying effectiveness, which include hollow tubes, scaffold-filled tubes containing neurotrophic factors, and those seeded with Schwann cells or stem cells [6, 10]. A number of experimental trials in animal models and some clinical cases demonstrated the efficacy of tubes from biomaterials in supporting peripheral nerve regeneration.

Self-assembling peptide (SAP) RARA16-I (Ac(RADA)_4_CONH_2_) is a synthetic amphiphilic peptide which undergoes spontaneous assembling in a controlled way into fibrils and eventually form a 3D hydrogel consisting of >99% water [11]. Such kind of peptide has attracted great interest in the field of nanotechnology for its potential for application in fields such as biomedical nanotechnology, cell culturing, molecular electronics and more. RARA16-I is synthesized by periodic repeats of alternating positively charged arginine (R), hydrophobic alanine (A), and negatively charged aspartic acids (D). Highly hydrate scaffold structures can be formed in the presence of physiological salt solution [12]. RADA16-I has been widely used in nerve repair according to its novel advantages: (i) good integration with different shapes of wounds [13]; (ii) high biocompatibility with low cytotoxicity inside the body [14]; (iii) a true 3D nanofibrous structure for cell growth [15, 16]. RADA16-I can be further modified with various functional motifs aiming at better bioactive performance [15, 17]. Several material scientists exploited RADA16-I as carrier for small molecules or proteins, the diffusion of which could be controlled by engineering RADA16-I with different motifs [18–20]. Therefore, SAP RADA16-I has been proving to be a promising platform for a variety of regenerative medicine applications.

However, there exists a significant limitation involved in the application of RADA16-I, which is the acidity of RADA16-I solution. Prior to hydrogel formation, the pH value of RADA16-I solution is around 3–4, which should be neutralized before cell seeding or transplantation *in vivo* [17, 21]. Direct contact with RADA16-I solution leads to cytotoxicity and inflammatory responses, both of which limit the application of RADA16-I in regenerating medicine. Numerous efforts have been devoted to solve the problem by modifying RADA 16-I with additional peptide sequence, the effects, however, are still not satisfactory. Recently, we reported a strategy to prepare nanofiber hydrogels at neutral pH from two functional SAPs by conjugating short functional motifs of IKVAV (Ile-Lys-Val-Ala-Val) and RGD (Arg-Gly-Asp) to the parent molecule RADA 16-I [22]. RGD is highly related to adult axon outgrowth during pathfinding [23–25]. IKAVA selectively promote neuronal differentiation and cell adhesion, and inhibit the differentiation and adhesion of glial cells [26, 27]. The two SAPs were oppositely charged in aqueous solution at physiological pH by specially designing. A 3D nanofibrous hydrogel, defined as RADA 16-MIX, was formed when combining them together. The designer RADA 16-MIX nanofibrous hydrogel could support neural progenitor cells (NPCs)/stem cells (NSCs) 3D growth and differentiation as well as create a permissive environment for the regeneration of peripheral nerve system and central nerve regeneration.

In the present study, we focused on the application of RADA16-Mix nanofibrous hydrogel in promoting the recovery of peripheral nerve injury. RADA 16-I and RADA16-Mix were transplanted to bridge the rat sciatic nerve defect. The axonal regeneration, target muscular re-innervation and motor functional recovery were thoroughly assessed.

## Materials and methods

### Animal subjects and experimental groups

Adult Sprague-Dawley (SD) female rats (88 in total) weighing 220 ± 20 g (From the Laboratory Animal Unit, The University of Hong Kong) were used, of which 15 rats were selected randomly to the control group; the remaining were randomly divided into three conduit groups: (i) the RADA16-Mix group (*n* = 29); (ii) the RADA16-I group (*n* = 26); (iii) saline group (*n* = 18). Some rats in the RADA16-Mix (*n* = 4), RADA16-I (*n* = 4) and saline (*n* = 2) groups were euthanized due to severe autotomy. As designed, rats were sacrificed at three different time points (4-, 8- and 12-week) post-surgery.

### Preparation of nanofibrous hydrogel for transplantation

In order to facilitate the transplantation of SAPs hydrogels, a conduit was fabricated by electrospinning poly-L-(lactic acid) (PLLA, purchased from Polysciences Ltd.) onto a rotating metallic rod (diameter = 1.5 mm) attached to a rotating mandrel. All the electrospinning experiments were conducted were according to our previous study [28, 29]. The inner diameter of the resultant conduit was 1.0 mm with the thickness of 150 μm. The two designer SAPs, RADA 16-RGD and RADA 16-IKVAV (custom synthesized by American Peptide Company, Inc.), were diluted in Milli-Q water separately and adjusted to a neutral pH value using 1M Tris buffer. The final density of both solutions was 10 mg/ml. The RADA16-I (Ac-(RADA)_4_-CONH_2_, BD Biosciences, Cambridge, MA, USA) solution with a concentrating of 10 mg/ml was used directly without further treatment.

### Sciatic nerve injury model

**Figure 1.**
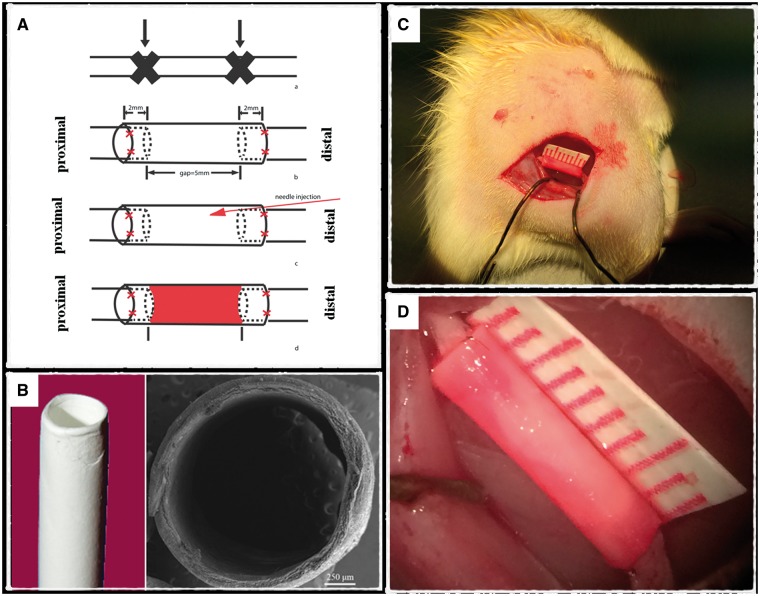
The schematic illustration of the experimental design (**A**) The injury model and repair strategy, (**B**) PLLA electrospun nanofibrous conduit. The scale bar in scanning electron micrograph is 250 μm. (**C**), (**D**) Surgery operated on rat right sciatic nerve. The graft was transplanted to bridge the nerve gap. The SAPs were injected into the conduit.

All rats were administered by a pre-anesthetic medication (buprenorphine, 0.05 mg/kg, 30 min before surgery), and then followed by anesthesia (Ketamine, 90 mg/kg, i.p.; Xylazine, 10 mg/kg, i.p.). The surgical operation commenced once an adequate level of anesthesia was achieved. After the right sciatic nerve was exposed, a 5.0 mm gap was made in the middle of the nerve trunk (Fig. 1) . The proximal cutting point was located at the vertical projection from the great trochanter to the sciatic nerve. For the RADA16-Mix, RADA16-I and saline groups, both the proximal and distal nerve stumps were inserted into the electrospun conduits (9 mm long) as far as 2.0 mm, and fixed with 10-0 suture (Ethicon). About 20 μl of either RADA16-Mix (immediately after mixing RADA 16-RGD and RADA 16-IKVAV neural solutions), RADA16-I or saline were then injected into the conduit with a 0.5 ml syringe with a 31 Gauge (BD Ultra-Fine II). After the injection, there was a 2-min wait to make sure that the RADA16-Mix/RADA16-I fully transformed into a hydrogel. For the control group, the transection was left without any treatment. The muscle was then closed with tissue glue (Histoacryl) and resutured the skin with wound clips (Michel Suture Clips; 11 × 2 mm). The rats were kept warm and allowed to recover from anesthesia. Antibiotics (Amoxicillin) and analgesic (Buprenorphine, Meloxicam and Acetaminophen) were used for post-operative care. In accordance with the guidelines for animal care and use published by The University of Hong Kong, the animals were allowed standard access to food and water *ad lib* throughout the study. In the event of severe autotomy, the rats were euthanized for ethical reasons.

### Histomorphometry of axonal regeneration

Animal subjects were sacrificed with sodium pentobarbital (160 mg/kg, i.p.); and then were perfused transcardially with 0.9% (w/v) saline and then with 4% paraformaldehyde (PFA) in 0.1 M phosphate buffer (PB). Involved right sciatic nerve segments, as well as the left side nerve, were harvested and followed by post-fixation in 4% PFA overnight at 4°C, and then immersed in 30% sucrose (w/v) in 0.1 M PB at 4°C until they sank to the bottom of the containers.

For the longitudinal section, the nerves with/without stumps were placed and transected longitudinally for 15 series of sections, and every 15th section was mounted on the same pre-coated slides. For the transverse section, three vertical lines were placed on the nerve stumps and conduit: two was 5 mm away from the nerve-conduit connection; and one in the middle of the conduit. Twenty series of 10 μm sections were collected and mounted on the pre-coated slides.

The series sections were selected randomly for immunohistochemical staining. After non-specific antigen binding was blocked for 1 h with blocking buffer (0.3% Triton, 5% Goat serum, 2% BSA in 0.01 M PBS) at room temperature; the sections were subsequently incubated with primary antibody solution overnight at 4°C. After washing with 0.01 M PBS, the sections were incubated with corresponding fluorescent-labeled secondary antibody at room temperature for 2 h. The primary antibody was rabbit anti-Neurofilament (NF) 200 (Sigma, 1:1000), which were used for labeling the neurofilaments. The corresponding secondary antibody was donkey anti-rabbit 568 (Invitrogen, 1:500). Finally, all the sections were mounted with the fluorescent mounting medium (Dako). Additionally, longitudinal sections from the 8- and 12-week groups were selected for routine Hematoxylin and Eosin staining.

Digital images were captured with a Zeiss-Axiophot microscope. Full view photos were merged with Adobe Photoshop CC software. To calculate the axonal numbers, three vertical lines were placed along the proximal–distal axis: proximal interface, midpoint and the distal junction site. The NF200-positive axons across theses vertical lines were quantified. (The total axonal number in one set of the section multiplied by the serial number (15) represented the axonal counts/animal.)

### Gastrocnemius muscle wet weight and muscular morphology

After the harvest of the sciatic nerve, the bilateral gastrocnemius muscles were dissected and weighed immediately. The weight ratio of injury side/intact side was calculated as the recovery index of gastrocnemius muscle. The unipennate gastrocnemius medialis (MGM) was dissect for cross cryosectioning with 10 μm thickness. Every 20th section was mounted on the pre-coated slides and double-immunostained with anti-α-bungarotoxin for motor endplate and anti-NF200 for neurofilament. Re-innervation was analysis by quantifying the overlapping neurofilament and α-bungarotoxin immunoreactivity. The re-innervation rate was calculated by the fully innervated motor endplates/total motor endplates.

### Behavioral test of motor function

The functional recovery of injured hind limb were assessed by Gait-Stance duration test preoperatively and every 4 weeks respectively post-treatment until experimental endpoint. The narrow runway was made of transparent plastic-bottomed box, consisting of a length of 36 cm, width of 6 cm and height of 7 cm. A darkened cage was connected at the end of the corridor to attract the animals. A high-speed digital image camera, recording at 120 frames per second, was positioned perpendicularly 40 cm under the center of the walkway. Animal subjects were trained to walk on the narrow runway at least four times before each assessment. There were three stages during the time period when the rat's foot was in contact with the floor: fist contact, weight support and take off. The duration time was regarded as the gait-stance duration (Walker *et al.* 1994 [31]). The ratio of right to left gait-stance duration was calculated as the functional recovery index for analysis.

### Statistical analysis

Statistical comparisons were performed using one way-ANOVA and Tukey's multiple comparisons test to analyze the differences among the groups. *P* < 0.05 was considered to represent a statistically significant difference.

## Results

### Axonal regenerative distribution and orientation within different grafts

In the current study, a peripheral nerve injury model was created by cutting and removing a 5-mm segment in the middle of the nerve trunk after the right sciatic nerve was exposed (Fig. 1). The electrospun PLLA nanofibrous conduit with an inner diameter similar to rat sciatic nerve was utilized to bridge the gap and simultaneously created an independent space for regenerating nerves. The conduit had a good mechanical property and could maintain the bulk conduit morphology without collapsing during the whole investigation period. The SAP nanofibrous hydrogels injected inside the conduit further provided a permissive environment to support regenerating nerves.

**Figure 2.**
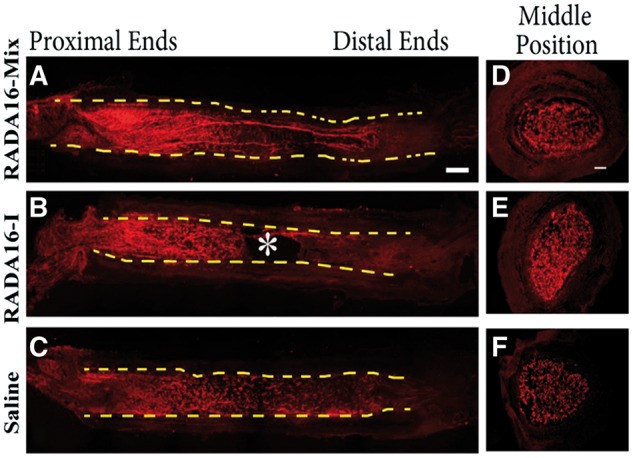
The longitudinal (**A**–**C**) and transverse (**D**–**F**) full views of axon regeneration 4 weeks after implanting different grafts (**A**, **D**) RADA16-mix, (**B**, **E**) RADA16-I group, (**C**, F) saline. The axons were labeled with rabbit anti-NF200 in red. The dash lines indicate the inside wall of the electrospun conduits. The * indicates of the large cavity formed inside the RADA16-I graft (**B**). Scale bar: 1000 μm.

Axonal regeneration occurred from the proximal nerve stumps as early as 4 weeks after surgery. The regenerated axons grew into the grafts and further crossed the lesion (Fig. 2). However, axons presented different morphologies within the three grafts. In the case of RADA 16-Mix graft (Fig. 2A), abundant axons aligned in the proximal ends of the injury site, whereas the axons were randomly dispersed in the proximal ends when RADA 16-I and saline were transplanted (Fig. 2B and C). Long axons were found to extend and further cross the whole lesion within the RADA 16-Mix. A large cavity was found in the RADA 16-I graft. The axons were only found to grow along the edge of the RADA 16-I graft, which was the wall of the electrospun conduit. Since saline would flow away soon after surgery, the connective tissues, cell products and scar entering from the two ends of the conduit fulfilled the conduit. It was thus difficult to form continuous and even structures, as evidenced by small cavities observed in the saline graft. Axon fragments were found to distribute in the fulfilling matters.

**Figure 3.**
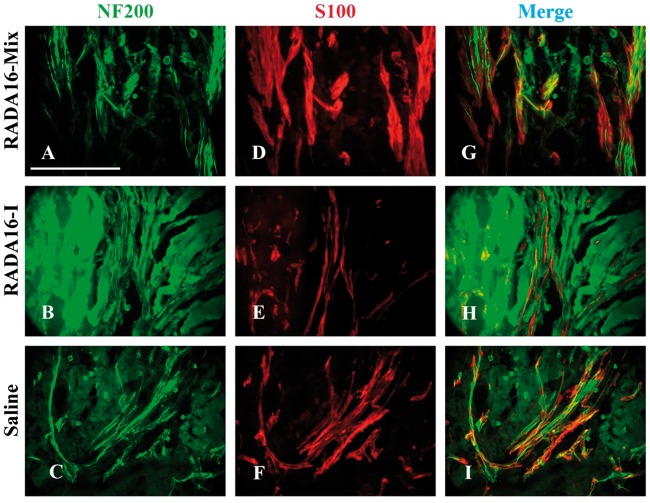
The longitudinal sections of the middle position 4 weeks after implanting different grafts. The axons were labeled by NF200 in green and Schwann cells were labeled by S100 in red. Scale bar: 200 μm.

Then we focused on the middle position of the grafts. As shown in Fig. 3, RADA 16-Mix and RADA 16-I displayed quite different structures. RADA16-Mix graft showed homogeneous porous structure while there were large cavities of long and narrow morphology in the RADA 16-I graft. Such differences were also found in our previous study when the two hydrogels were implanted into spinal cord [22]. There were axons regenerating and Schwann cell immigrating into the two SAP nanofibrous hydrogel grafts. The axons and Schwann cells were uniformly distributed within the RADA 16-Mix nanofibrous hydrogel while the axons and Schwann cells grew along the crevices of RADA 16-I bulk rather than within the bulk. There were also dense matters found to fill the electrspun conduit 4 weeks after saline injection (Fig. 3I). Similar to those in the RADA 16-I grafts, the axons and Schwann cells were found to grow along the crevices of the dense matters. Therefore, the distribution and orientation of regenerating axons strongly depended on the structure of SAP nanofibrous hydrogel.

**Figure 4.**
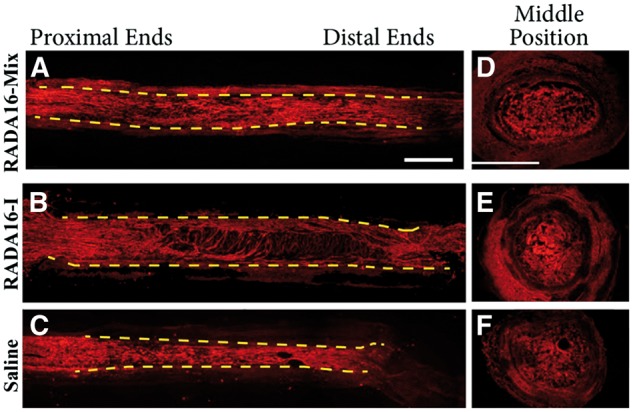
The longitudinal and transverse full views of axon regeneration 8 weeks after implanting different grafts (**A**) RADA16-mix, (**B**) RADA16-I group, (**C**) saline. The axons were labeled with rabbit anti-NF200 in red. The dash lines indicate the inside wall of the electrospun conduit. Scale bar: 1000 μm.

Eight weeks after transplantation, more axons regenerated within all the three grafts. However, the regenerating axons still exhibited different distribution and orientation as shown in Fig. 4A. Similar to those 4 weeks after transplantation, the axons were uniformly dispersed within the RADA 16-Mix after 8 weeks. The axons grew towards the distal end in a parallel arrangement to the direction of the conduit. Although the axons were in a parallel arrangement in the proximal end, they became random with growing and oriented in the radial direction of the electrospun conduit within the RADA 16-I hydrogel as indicated by the arrow. Axons also grew toward the distal end in the electrospun conduit, but several large cavities were found. Moreover, conduit with saline injection was thinner than those were injected with RADA 16-Mix and RADA 16-I hydrogels, probably because of the pressure from surrounding tissues. The transverse section of the RADA 16-Mix graft at the middle position presented uniform porous structure and corresponding axon distribution. However, when transplanted with RADA 16-I hydrogel and saline, dense bulk and large cavities were observed in the transverse section, in good agreement with the longitudinal view of the grafts.

**Figure 5.**
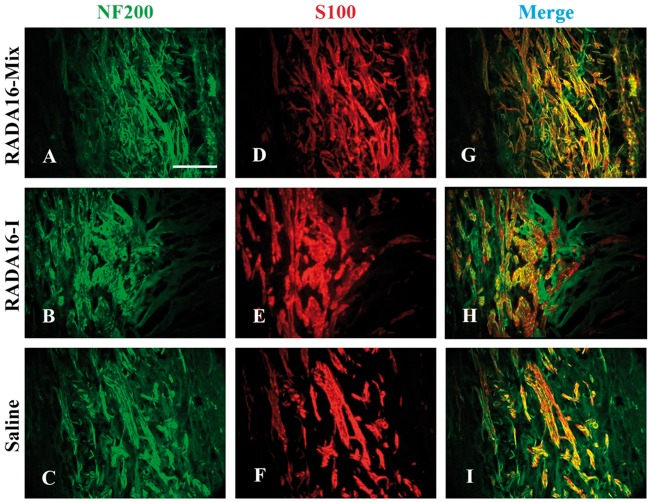
The longitudinal sections of the middle position 8 weeks after implanting different grafts. The axons were labeled by NF200 in green and Schwann cells were labeled by S100 in red. Scale bar: 200 μm.

After 8 weeks, the axons and Schwann cells exhibited a similar distribution and orientation within the grafts to those after transplantation for 4 weeks (Fig. 5). The axons and Schwann cells were uniformly distributed within RADA 16-Mix grafts, while those grew along the crevices of RADA 16-I bulk rather than within the bulk.

**Figure 6.**
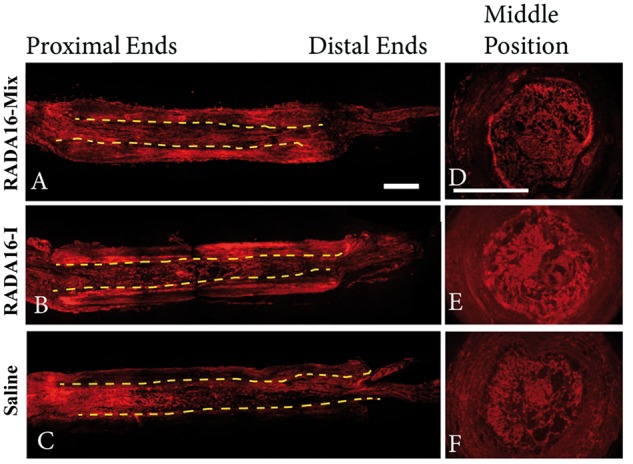
The longitudinal and transverse full views of axon regeneration 12 weeks after implanting different grafts (**A**) RADA16-mix, (**B**) RADA16-I group, (**C**) saline. The axons were labeled with rabbit anti-NF200 in red. The dash lines indicate the inside wall of the electrospun conduit. Scale bar: 1000 μm.

**Figure 7.**
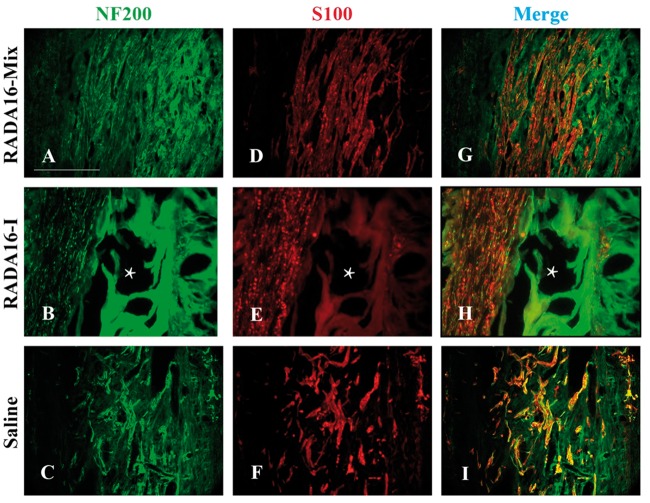
The longitudinal sections of the middle position 12 weeks after implanting different grafts. The axons were labeled by NF200 in green and Schwann cells were labeled by S100 in red. The * shows the large cavity formed inside the RADA16-I graft. Scale bar: 200 μm.

Figure 6 presented the longitudinal and transverse full views of the grafts after transplantation for 12 weeks. The electrospun conduit was filled with the parallel axons along the axial direction within the RADA 16-Mix hydrogel (Fig. 6A and D). Parallel axons were observed within the RADA 16-I hydrogel after transplantation for 12 weeks, axons of random distribution and those orienting in the radial direction of the electrospun conduit were observed. Large cavities and dense RADA 16-Mix hydrogel bulk (Fig. 6E)/matters remained (Fig. 6F) within the electrospun conduit. Axons and Schwann cells remained the distribution within the three grafts even after 12 weeks, as indicated by Fig. 7. The axons and Schwann cells were uniformly distributed within RADA 16-Mix hydrogel graft while seldom axons and Schwann cells were observed to grow within RADA 16-I hydrogel bulk. Moreover, larger cavities were observed in the RADA 16-I hydrogel grafts as compared to those after 4 and 8 weeks, which might be due to RADA 16-I degradation (Fig. 7).

**Figure 8.**
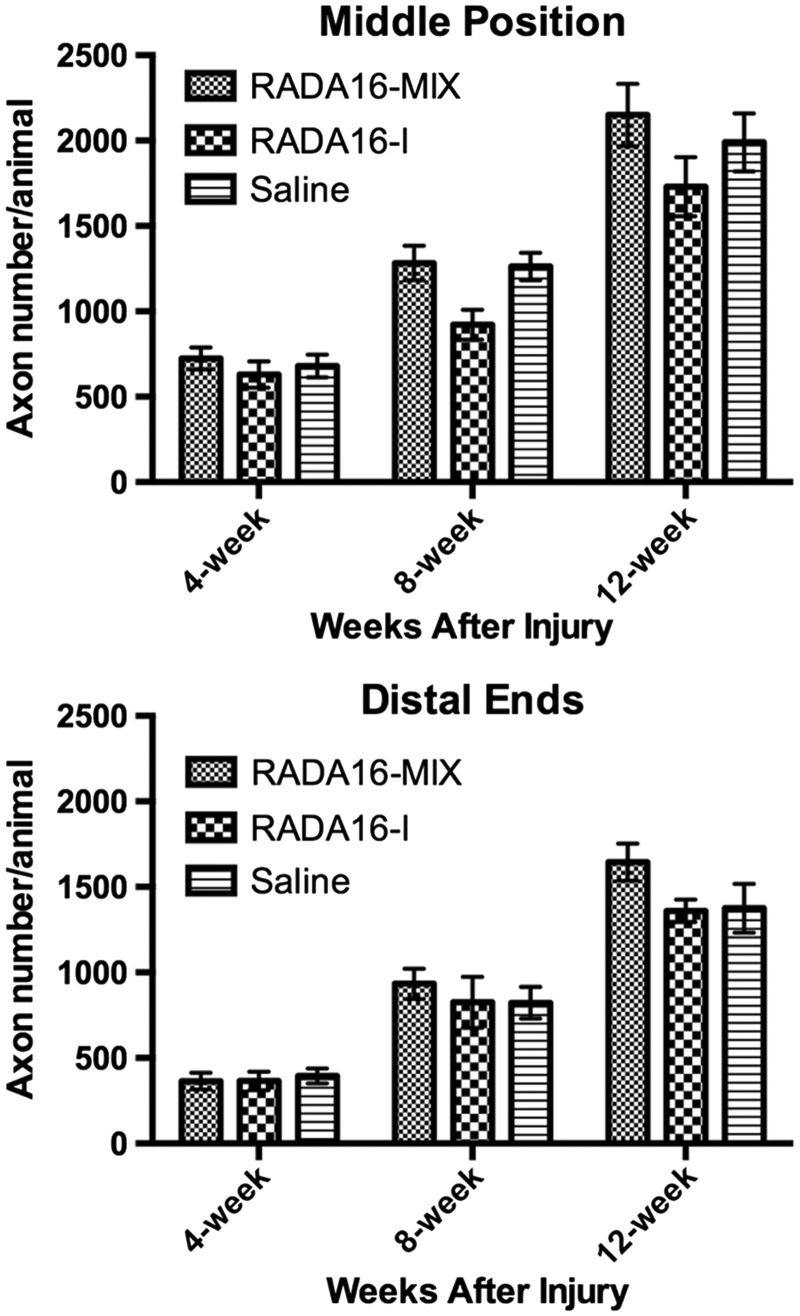
The axon numbers at the midpoints and distal planes after different transplantation time (one-way ANOVA test and Tukey’s multiple comparisons test; *P* > 0.05).

The number of axons is an important index for nerve regeneration, including the entrance, cross and exit the graft. Since the regenerating axons in the proximal end of the three grafts were much similar, we mainly focused on comparing the numbers of axons regenerated across the midpoints and distal planes. In the midpoint and distal planes, the axon number increased with transplantation time (Fig. 8). Four weeks after transplantation, the axon number in the middle position of RADA16-Mix graft was slightly higher than those in the RADA16-I graft and saline though there was no significance. After 8 and 12 weeks, much more axons regenerated and crossed the middle position of RADA16-Mix graft than RADA16-I graft. Interestingly, RADA16-Mix graft and saline had similar axon number. However, the axons exited the RADA16-I graft and saline had similar number 8 weeks after transplantation, smaller than those exited the RADA16-Mix graft. Especially after 12 weeks, significantly more axons exited the RADA16-Mix graft than RADA16-I graft and saline and 12 weeks, indicating the advantages of RADA16-Mix nanofibrous hydrogel over RADA16-I in supporting axons regrowth.

**Figure 9.**
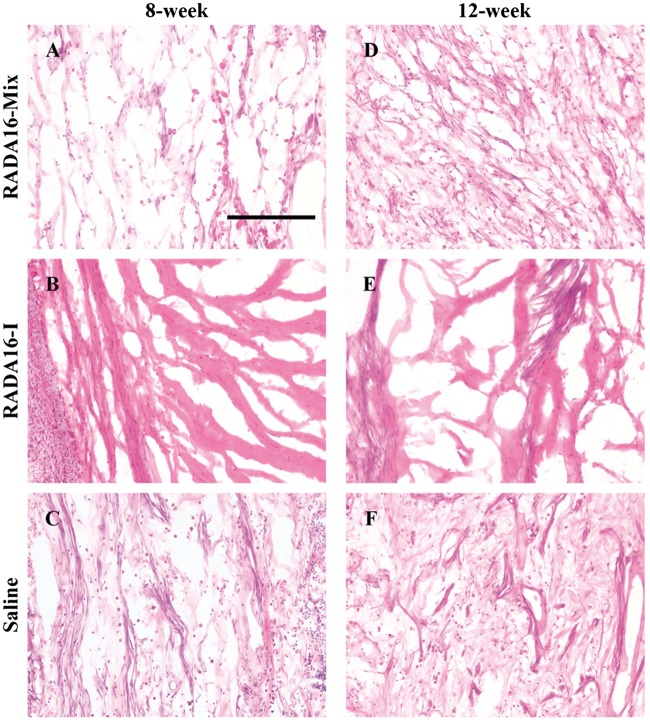
The Hematoxylin and Eosin staining of the midpoint of the grafts. Scale bar: 200 μm.

HE staining was performed 8 and 12 weeks after transplanting RADA 16-I, RADA 16-Mix and saline with the results shown in Fig. 9. There were cells immigration into the grafts and more cells were observed with longer transplantation time. The recruited cells were uniformly distributed in the homogenous RADA 16-Mix hydrogel while fewer cells were distributed within the RADA 16-I hydrogel bulk. There were large cavities in the RADA 16-I graft 8 and 12 weeks after transplantation, which was in good agreement with aforementioned immunochemical staining analysis. As mentioned above, dense matters were observed to fill the electrospun conduit injected with saline which might provide support for the regenerating axons. Numerous cells were observed in the matters. Therefore, transplantation with the electrospun hollow conduit could support axon regeneration and extension via the scaffold formed by the connective tissues, scars and cellular products.

### Formation of new neuromuscular junction structures

**Figure 10.**
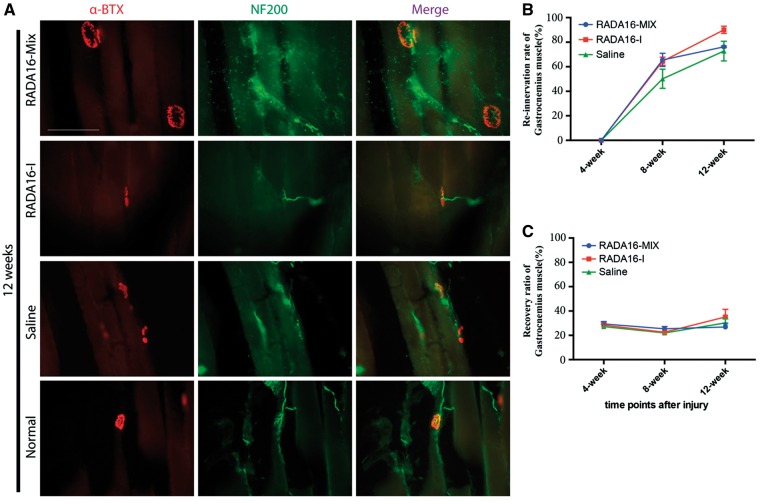
The formation of new NMJ structures 12 weeks after transplantation, scale bar: 100 μm (**A**) the re-innervation rate, (**B**) and the weight ratio (**C**) at different transplantation time.

The formation of new neuromuscular junction (NMJ) structures plays a critical role in functional recovery. In this study, the immunostaining with anti-α-bungarotoxin (a-BTX) for motor endplate and anti-NF200 for neurofilament were simultaneously performed. As shown in Fig. 10A, NF200 immunostaining was coincident with the a-BTX binding sites in the muscles of normal rats. Similar NMJ structures were detected in all the muscles with the three grafts 12 weeks after transplantation. However, atrophy of the motor endplate in the case of saline graft was significantly serious and the NMJ structures were noticeably sparser as compared with those in the cases with RADA 16-Mix and RADA 16-I hydrogel grafts, indicating the significance of SAP nanofibrous hydrogel transplantation. The re-innervation rate was calculated by the number of the fully innervated motor endplates divided by the number of the total motor endplates (Fig. 10B). No re-innervation found at 4 weeks post-surgery and the re-innervation rate was above 60% for both RADA 16-Mix and RADA 16-I grafts after 8 weeks, higher than that of saline. Twelve weeks after transplantation, RADA 16-I hydrogel exhibited highest re-innervation rate among the three grafts but displayed no statistical significance. The muscular recovery after injury was investigated by measuring the gastrocnemius muscle wet weight as the recovery index of gastrocnemius muscle (Fig. 10C). The weight ratio of injured/intact side showed a slight decrease with transplantation time and then increased after 12 weeks, which might be attributing to re-innervation. Similarly, there were no statistically significant differences among the three transplantation interventions.

### Functional recovery investigation

**Figure 11.**
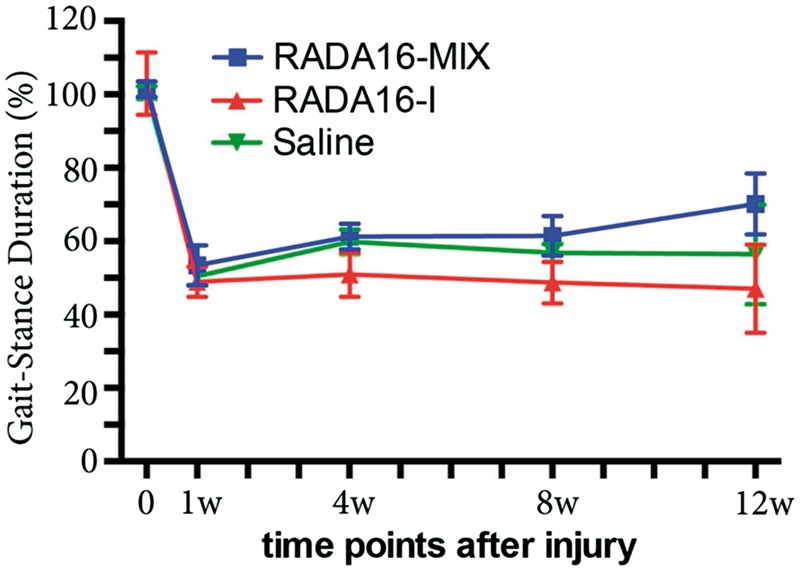
Functional recovery as determined by the technique of gait-stance duration (one-way ANOVA test and Tukey’s multiple comparisons test; *P* > 0.05).

The technique of gait-stance duration was utilized to evaluate functional recovery after sciatic nerve injury by calculating a ratio of injured/uninjured hind feet [30]. The gait-stance duration percentage (GSD%) calculated by comparisons to normal gait was presented in Fig. 11. GSD% decreased immediately after injury followed by slight increase after transplanting RADA 16-Mix graft and increased to around 70% after 12 weeks. In contrast, GSD% continued to decrease since the fourth week after transplanting RADA 16-I and saline grafts although there was a slight increase as compared to the first week. Therefore, RADA 16-Mix once again showed advantages over RADA 16-I in promoting peripheral nerve function recovery after injury.

## Discussion

During the last decades significant progress has been made in understanding the physiopathology of the peripheral nerve injury and regeneration. Compared with the hostile nature of central nervous system (CNS) to axonal regeneration, peripheral nervous system (PNS) provides relatively permissive environment to axonal regrowth. However, the outcomes of regeneration following injury remain frustratingly poor due to slow, insufficient and misdirected axonal outgrowth at the site of injury as well as atrophy of muscle tissue and failure of re-innervation at the target organ [6]. At the present time, the challenge is to find alternative treatments to the nerve graft and to improve recovery rates as well as functional outcomes [6, 10]. The current strategies mainly focused on developing permissive substrates for axonal regeneration. RADA16-I is a well-documented SAP that can form nanofibrous hydrogel with the structure mimicking natural extracellular matrix. But its acidic nature prior to hydrogelation is toxic to cells and tissues, causing inflammations as well as necrosis. To avoid this disadvantage, in the previous study, we modified the RADA16-1 to reach a neutral pH value. In addition, two functional motifs, IKVAV and RGD, which facilitate neuritis outgrowth and attachment, were added into its molecular chain. Such modified SAP hydrogel, named RADA16-Mix was proven to support NSCs/NPCs 3D growth and differentiation *in vitro*, as well as provided a permissive environment for nerve regeneration after central nerve injuries [22, 31].

In the current study, we investigated its application in promoting peripheral nerve regeneration. The different process of hydrogel formation might have contributed to the differences in the structure of RADA16-Mix and RADA16-I after hydrogelation. The RADA16-I hydrogel underwent a self-assembling process initiated by increasing the pH to 7 or exposure to salt solutions. Therefore, incubation with a large amount of neural medium was required immediately in order to equilibrate the RADA16-I solution to physiological pH *in vitro* [21]. When it was utilized for *in vivo* injection, RADA16-I gelation was initiated by the influx body fluid. The rapid rate at which the flow of influx body fluid was arrested suggested that only a small amount of nanofibers closely associated with the tissue were formed. Thus the time scale was too short to achieve full assembly of the peptide, leading to heterogeneous structure and large cavities within the resultant hydrogel. In the contrast, the self-assembly of RADA16-Mix was induced by electrostatic interactions between the two oppositely charged SAPs, giving rise to homogeneous structure when two peptides were mixed. That’s why RADA 16-Mix exhibited homogenous structure after transplantation in the conduit bridging the gap of the transected sciatic nerve while RADA 16-I graft was composed of the hierarchical overlapping dense bulk with long and narrow cavities along the radial direction of the conduit. Since the topography of the substrate greatly influenced the neurite outgrowth [29], the regenerating axons exhibited different distribution and extension within the hydrogel bulk. In the RADA 16-Mix hydrogel graft, the axons grew in parallel rows along the axial direction of the electrospun conduit. In contrast, the axons in the RADA 16-I hydrogel graft extended along the wall surfaces of the cavities, seldom axons were found to grow into the dense hydrogel bulk. Moreover, most of the cavities were closed with the long axis of the cavities perpendicular to the conduit. Overall, the axons grew around the RADA 16-I hydrogel graft rather than growing into the SAPs, or arranged in the direction perpendicular to the conduit in spite of highly parallel axons in the proximal nerve stumps. Such observation was consistent to the results observed in central nerve regeneration in the previous study [22, 31]. Therefore, the RADA 16-Mix SAP nanofibrous hydrogel provided a permissive environment with better structure for peripheral nerve regeneration than RADA 16-I. As a result, more axons regenerated in the RADA 16-Mix graft than those regenerated in RADA 16-I graft. In our results, we also found that nerves were able to regenerate into the hollow electrospun conduit in the saline group. The conduit might serves as a scaffold for fibroblasts or Schwann cells to migrate to and assembly along the wall of conduit, providing support for axonal regrowth. This phenomenon is in agreement with our previous study [28]. But compared with RADA 16-Mix group, the number of regenerated axons was still limited. These results suggested that RADA 16-Mix outperformed RADA 16-I and saline in supporting axonal regeneration.

However, in the distal part of the transected nerve, RADA 16-Mix group failed to show significantly more regenerated axons compared with other two grafted groups, even with the control. This result was paralleled with the following analysis of re-innervated endplates after grafting and behavior test. In both study, RADA 16-Mix group failed to display any significant advantage over other groups, indicating that although RADA 16-Mix outperformed RADA 16-I and saline in supporting axonal regeneration, it is still not sufficient to let axons grow to the distal part of transected nerve to re-establish NMJ. Other promotions might be combined. The regeneration of nerve is a complex process; yet, a single approach on its own has not allowed an effective therapy. Advances in the areas of drug/gene delivery and cell-based therapies have greatly promoted the development of peripheral nerve injury repair. Biomaterials of good biocompatibility as well as structural and mechanical performances can provide supportive carrier for drugs, genes, cells and the regenerating axons. Therefore, the functional SAP RADA 16-Mix has a potential application in enhancing axonal growth and increasing the recovery of functions in the nerve system in combination with delivery system strategy in the future.

## Conclusion

In this study, we prepared a functional SAP nanofibrous hydrogel by modifying RADA 16-I and investigated its application in promoting peripheral nerve regeneration. As compared with RADA 16-I, RADA 16-Mix SAP nanofibrous hydrogel provided a better environment for rat sciatic nerve regeneration. Combing with our previous studies on the application of RADA 16-Mix nanofibrous hydrogel in promoting central nerve regeneration, the designer RADA 16-Mix SAP has shown great potential in nerve regeneration and injury repair.
